# Evaluating the Cost-Effectiveness of Proportional-Assist Ventilation Plus vs. Pressure Support Ventilation in the Intensive Care Unit in Two Countries

**DOI:** 10.3389/fpubh.2018.00168

**Published:** 2018-06-06

**Authors:** Rhodri Saunders, Dimitris Geogopoulos

**Affiliations:** ^1^Coreva Scientific, Freiburg, Germany; ^2^Medical School, University Hospital of Heraklion, University of Crete, Heraklion, Greece

**Keywords:** artificial respiration, critical care, cost-benefit analysis, health care costs, quality of life

## Abstract

**Background:** Mechanical ventilation is an integral, but expensive, part of the intensive care unit (ICU). Optimal use of mechanical ventilation could save costs and improve patient outcomes. Here, the cost effectiveness of proportional assist ventilation (PAV™ ventilation by Medtronic) is estimated relative to pressure support ventilation (PSV).

**Methods:** A cohort-level, clinical model was built using data from clinical trials. The model estimates patient-ventilator asynchrony >10%, tracheostomy, ventilator-associated pneumonia, other nosocomial infections, spontaneous breathing trial success, hypoxemia, and death. Cost and quality of life are associated with all events, with cost effectiveness defined as the cost per quality-adjusted life year (QALY) gained in the US and UK.

**Results:** The mean cost of ICU care was lower with PAV™ than with PSV in the US and UK, but the total cost of care over 40 years was higher due to more patients surviving and incurring future care costs. Reduced time on mechanical ventilation, fewer nosocomial infections, and extended life expectancy with PAV™ drove QALY improvement. The cost per QALY gained with PAV™ was $8,628 and £2,985.

**Conclusion:** PAV™ improves quality of life and reduces short-term costs. PAV™ is likely to be considered cost-effective over 40-years in the US and UK.

## Background

Mechanical ventilation via an endotracheal tube, is life-saving for patients with acute respiratory failure in the intensive care unit (ICU). As the most widely used supportive technique in the ICU (1), its patient benefit is generally accepted. It is, however, an invasive and expensive intervention. Uncomplicated mechanical ventilation in the US was found to have a mean cost of $59,770 per patient in 2009 USD (2). European studies have determined that the daily, direct costs of an ICU stay range between €1,168 to €2,025 (3, 4), with UK costs reported at £1,738 in 2016 (5). The patient impact also cannot be ignored, with studies showing that patients in critical care have a negative (worse than death) quality of life (6). As it is expected that mechanical ventilation will remain an integral part of care for years to come (7), understanding the most beneficial modes of mechanical ventilation from both a patient and healthcare provider point of view is of increasing importance.

There are various modes of mechanical ventilation, which can be generally split into “controlled” and “assisted.” With controlled modes the respiratory system is a passive structure and all breath characteristics depend on ventilator settings as determined by the caregiver and respiratory system mechanics (8). During assisted modes of support, the patient's control of breathing is under the influence of the ventilator pump and the ventilatory output is the final expression of the interaction between the ventilator and the patient's system of control of breathing (9). With common modes of assisted mechanical ventilation, such as volume-assist and pressure support ventilation (PSV), the algorithms used to deliver pressure and cycle off the ventilator are far from ideal and patient-ventilator asynchrony occurs regularly. This is not without consequences, since several studies have shown that increased asynchrony, particularly ineffective efforts, is associated with poor outcome (10–13).

Proportional-assist ventilation with load-adjustable gain factors (PAV™, Medtronic Inc.) is an assisted mode in which ventilator software measures, semi-continuously, elastance and resistance of the respiratory system and once triggered delivers pressure proportional to the instantaneous inspiratory flow and volume and, hence, to the inspiratory muscles' pressure (14). Several studies demonstrated that, compared to PSV, PAV™ improves patient–ventilator synchrony and unloads the respiratory muscles without the risk of over-assistance and periodic breathing (15–17). Whether or not PAV™ constitutes a cost-effective approach to care in the ICU remains, though, an open question.

The costs and patient outcomes, in particular mortality, related to mechanical ventilation are realized over years and decades (18, 19). This makes an evaluation of cost-effectiveness as part of a real-life study impractical. The analysis described here explores the question of cost-effectiveness using a computational model of the patient care pathway and is applied to the United States (US) and United Kingdom (UK) settings. The *in-silico* approach taken complies with good practice guidelines and the requirements of health technology assessment agencies (20, 21).

## Methods

This analysis considers a cohort of patients receiving mechanical ventilation in the ICU and their progression through the care pathway to hospital discharge. Using clinical outcome data from randomized, controlled and large, prospective studies, an *in-silico* model of the care pathway was developed. Each clinical setting and event was associated with a cost and patient quality-of-life utility. Clinical outcome, cost, and quality-of-life data were sourced from a structured review of peer-reviewed literature. Searches of PubMed were performed on January 5, 2017 to identify recent data related to the critical care setting, healthcare costs, quality of life utilities, and efficacy and safety of PSV and PAV™. The structured searches are available in the Supplementary Material.

The developed Markov model (Figure S1 in Supplementary Material) has patients (Table 1) starting in the ICU and receiving either PSV or PAV™. At initiation of mechanical ventilation, 38% of patients were found to have clusters of ineffective ventilatory efforts (or ventilator asynchronies), which were linked to a high asynchrony index (13). In 8.4% of cases, patients with clusters of ineffective efforts exhibited an asynchrony index >10% (13). In comparison, only 1.5% of patients without clusters of ineffective efforts had an asynchrony index >10% (13). Asynchrony >10% is associated with additional time in the ICU and increased risk of tracheotomy (11). At each time point in the model, patients are assessed for asynchrony ≤10%, asynchrony >10%, tracheostomy, ventilator-associated pneumonia (VAP), other nosocomial infection, and a spontaneous breathing trial (SBT). Patients who failed on a SBT remain on mechanical ventilation and may experience hypoxemia; whereas, if successful the patient may be weaned and is eligible for transfer to the general ward (17). Patients remain on the general ward until discharge home. Mortality rates in the ICU, in hospital, and after discharge home are taken from published literature specific to this patient population (18, 19).

**Figure 1:**
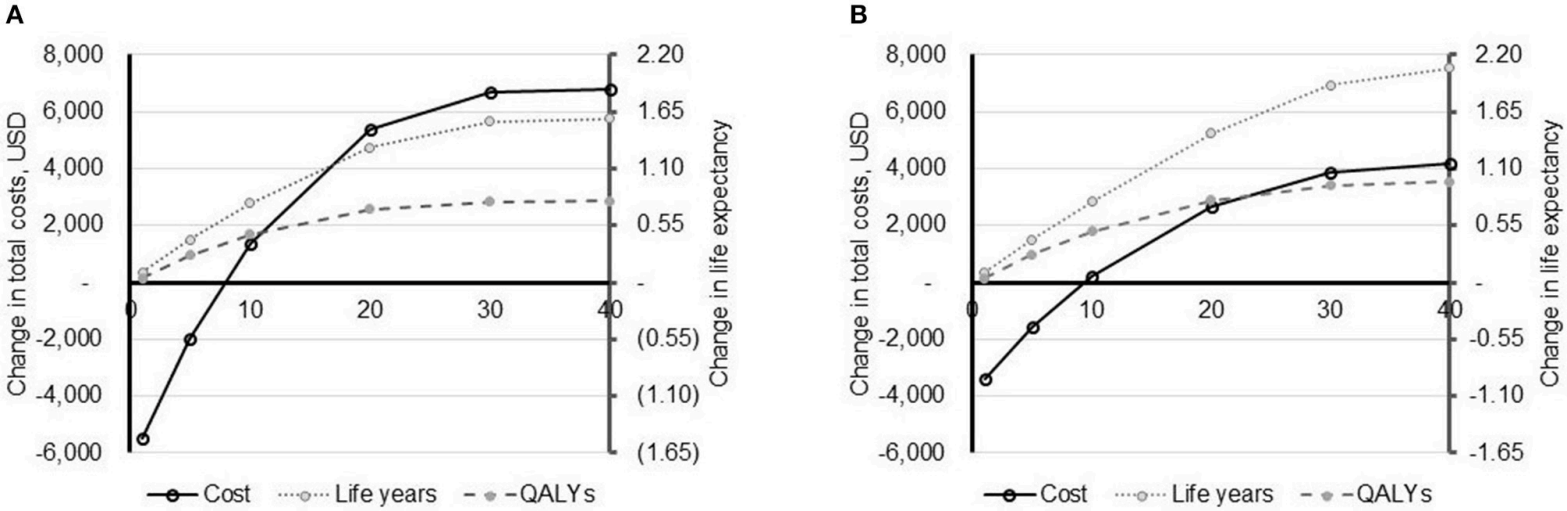
Impact of time horizon on model outcomes. The impact of the time horizon (X-axis, years) on model outcomes: total costs (primary Y-axis) and life expectancy (secondary Y-axis) is depicted for the US **(A)** and UK (costs converted to USD to allow comparison) **(B)**. Life expectancy is shown in both life years and quality-adjusted life years (QALYs). All data are reported as PAV™ minus PSV.

**Table 1:** Model parameters by country setting.

**Model parameter**	**US**	**UK**
Mean (SD) age, years	63.5 (16.3) (22)	55.4 (16.8) (5)
Gender, % female	43.9 (22)	37.7 (5)
**EVENTS**		
VAP, %	4.4 (after 10 days) (19)	15.2 per 1,000 days) (23)
Other nosocomial infection, %	0.85 (after 1 day) (24)	0.85 (after 1 day) (24)
Tracheostomy, %	13.9 (after 10 days) (25)	14 (after 12 days) (19)
COSTS, IN 2015 USD ($) OR GBP (£)^*^		
Mechanical ventilation, initiation	$881 (22)	£200^†^ [$294]
Mechanical ventilation, per day	$7,074 (22)	£1,863 (5) [$2,736]
ICU stay, per day	$4,818 (22)	£1,863 (5) [$2,736]
Hospital stay, per day	$2,271	£785 (5) [$1,153]
Post-discharge (home), per year	$12,189 (26)	£4,218 (5) [$6,194]
Tracheostomy, per event	$1,017 (27)	£574 (27) [$843]
Hypoxemia, per event	$10^†^	£10^†^ [$15]
VAP, per day	$3,192 (28)	£61 (29) [$90]
Other nosocomial infection, per event	$933 (28)	£572 (30) [$840]
**QUALITY OF LIFE**		
Baseline	0.776 (31)	0.804 (31)
Annual disutility	0.003 (31)	0.003 (31)
Mechanical ventilation	−0.39 (6)	−0.39 (6)
ICU	0.40 (32)	0.40 (32)
Hospital	0.52 (5)	0.52 (5)
First post-discharge year	0.55 (5)	0.55 (5)

*GBP, Great Britain Pounds Sterling; ICU, Intensive care unit; SD, Standard deviation; UK, United Kingdom; US, United States; USD, United States Dollar; VAP, Ventilator-associated pneumonia. USD values in square brackets are for comparative purposes and calculated using the mean annual exchange rate for USD:GBP in 2015*.

* *Values in the cited reference may vary from those presented due to adjustment to 2015 pricing using the Producer Price Index for General medical and surgical hospitals (PCU622110622110) in the US setting and the CPIH INDEX 06 (L528) released March 14, 2017 in the UK setting*.

† *Assumed cost, no data identified*.

*^‡^The Kaiser Family Foundation State Health Facts. Data Source: Hospital Adjusted Expenses per Inpatient Day. Data located at http://kff.org/health-costs/state-indicator/expenses-per-inpatient-day/?currentTimeframe=0&sortModel=%7B%22colId%22:%22Location%22,%22sort%22:%22asc%22%7D and last accessed July 21, 2017*.

The relative efficacy of PAV™ compared with PSV was taken from published, randomized controlled trials (16, 17). From their pilot study, Bosma et al. reported that PAV™ resulted in a significant reduction in time on mechanical ventilation and in the ICU, but was associated with an extended stay in hospital (Table 2) (16). The authors also presented information on the number of patients experiencing asynchrony and receiving a tracheostomy tube, both of which were lower with PAV™ than with PSV (16). The study by Xirouchaki et al., determined that PAV™ was associated with increased probability on remaining on assisted modes (17). Other endpoints in this study were numerically lower with PAV™ but the difference did not reach significance (17).

**Table 2:** Model parameters by method of mechanical ventilation.

**Model parameter**	**PSV**	**PAV™**
Asynchrony >10%, % of days in state	26.3 (16)	7.9 (16)
MV time, days	5 (19)	−1.0, (16) relative to PSV
ICU time, days	7 (19)	−5.1, (16) relative to PSV
Hospital time, days	17 (19)	+1.5, (16) relative to PSV
Tracheostomy, %	Setting specific (Table 1)	RR 0.57 (0.18; 1.77) (16)
SBT success, %	77.9 (after 2 days) (17)	RR 1.14 (1.01;1.29) (17)
Remain on ventilator after SBT success, %	54 (17)	RR 0.86 (0.65; 1.13) (17)
Hypoxemia if failed SBT, %	45.5 (17)	RR 0.55 (0.19; 1.62) (17)
**MORTALITY…**		
…in ICU, %	28 (after 14 days) (19)	RR 0.76 (0.44; 1.32) (17)
…in hospital, %	35 (after 31 days) (19)	RR 0.72 (0.23; 2.25) (17)
…after discharge, %		
Year 1, %	12.5 (18)	12.5 (18)
Year 2, %	19.3 (18)	19.3 (18)
Year 3, %	27.5 (18)	27.5 (18)
Year 4 onwards	National life tables	National life tables

*ICU, Intensive care unit; MV, Mechanical ventilation; PAV™, proportional-assist ventilation with load-adjustable gain factors; PSV, Pressure support ventilation; RR, Relative risk; SBT, Spontaneous breathing trial. RR are reported as the mean (95% confidence interval)*.

For each event, a cost (in 2015 currency units) and quality of life utility is applied (Table 1). For example, VAP is known to increase the cost of care and the time on mechanical ventilation, adding about 11 days (2, 33), and likely resulting in reduced patient quality of life through increased time in the ICU. In addition, the interventions (PSV or PAV™) also have a cost, which were assumed to be equivalent at USD 27,000 or GBP 27,000 for purchase. Assuming a 5-year life cycle and usage of 80% of days, the cost per day for the each intervention was USD/GBP 18.48. The summation of costs and quality of life over the time horizon of the model allows for the cost effectiveness of mechanical ventilation methods to be assessed. The model was developed in line with ISPOR good practice guidelines and includes all relevant clinical aspects for which incidence data were available, irrespective of whether efficacy data for the comparators or any cost or quality of life data were available (34). The time line for analysis is 40 years, and provides a healthcare payer perspective of expected costs over the lifetime of the patient. All costs and utilities were discounted at 3.5% per annum after year 1. Results are reported as the cost per quality-adjusted life year (QALY) gained with PAV™ relative to PSV. To facilitate comparison, UK costs are also converted to 2015 USD using the mean annual exchange rate (1 USD = 0.68 GBP^1^).

The model outcome in the base case provides an estimate of the costs and QALYs associated with use of PSV and PAV™. There is, though, inherent uncertainty about the parameters used as model inputs, which is often described via the standard deviation or 95% confidence interval. To understand how the uncertainty in model inputs influences outcomes, 2,000 patient cohorts were simulated using sampled age and gender distributions and run through the model. In each case, all model parameters (such as incidence of VAP and the relative risk of a successful SBT with PAV™) were also sampled from underlying distributions. Incidence data used normal, and relative risks log-normal, distributions. The 2,000 analyses were also used to estimate the cost-effectiveness plane and the likelihood of PAV™ being considered cost effective. The willingness-to-pay threshold, the maximum level at which a payer would consider an intervention to be cost effective, was taken to be the commonly accepted values of USD 50,000 and GBP 30,000 per QALY gained in the US and UK, respectively (35).

As model outcome data rarely follow a normal distribution, frequentist statistics and their calculated *p*-values are generally not appropriate measures of significance. As such, the 95% credible interval (CrI) for outcomes of the 2,000 simulations were also calculated. This is the outcome range, in which 95% of results fall (e.g., the bottom and top 2.5% of results are excluded). Although no direct relationship holds between the Bayesian CrI and the frequentist confidence interval, a 95% CrI that does not cross parity can be approximated to the same significance as a *p*-value of < 0.05.

## Results

Over 40 years in the base case compared with PSV, PAV™ was associated with increased life expectancy and quality-adjusted life expectancy (Table 3). In the US setting, the increases were 1.58 years (10.53 vs. 12.11) and 0.79 QALYs (5.21 vs. 6.00), whereas in the UK setting PAV™ resulted in an extra 2.05 years (13.85 vs. 15.90) and 0.97 QALYs (6.52 vs. 7.48). The increase in QALYs with use of PAV™ will in part be due to fewer days on mechanical ventilation and a reduction in days with VAP (21.7 and 22.0% reduction in the US and UK, respectively). There were also fewer nosocomial infections and tracheostomies with PAV™ (Table 3). In both the US and UK, these beneficial outcomes were associated with higher costs. The increase was USD 6,805 (USD 170 per year of the model) in the US and GBP 2,891 [$4,245] (GBP 72 [$106] per year) in the UK. The incremental cost-effectiveness ratios (ICER), USD 8,628 per QALY gained in the US and GBP 2,985 [$4,383] per QALY gained in the UK, were substantially below the willingness-to-pay threshold in each country.

**Table 3:** Base case outcomes for PSV and PAV™.

**Outcome**	**US: PSV**	**US: PAV™**	**US: Difference**	**UK: PSV**	**UK: PAV™**	**UK: Difference**
Total cost	$141,848	$148,653	+$6,805	£56,462 [$82,910]	£59,352 [$87,154]	+£2,891 [$4,245]
Life expectancy, years	10.53	12.11	+1.58	13.85	15.90	+2.05
QALYs	5.21	6.00	+0.79	6.52	7.48	+0.97
MV days, mean	6.97	5.43	−1.54	6.77	5.26	−1.51
Tracheostomy, %	8.5	3.7	−4.8	7.0	3.0	−4.0
Nosocomial infections, %	10.1	9.8	−0.3	9.9	9.7	−0.2
**ICER**						
Cost per life year gained	–	$4,297	–	–	£1,412 [$2,073]	–
Cost per QALY gained	–	$8,628	–	–	£2,985 [$4,383]	–

*ICER, Incremental cost-effectiveness ratio; MV, Mechanical ventilation; PAV™, proportional-assist ventilation with load-adjustable gain factors; PSV, Pressure support ventilation; QALY, Quality-adjusted life year; UK, United Kingdom; US, United States. Differences are reported as PAV™ minus PSV. USD values in square brackets are for comparative purposes and calculated using the mean annual exchange rate for USD:GBP in 2015*.

In varying the time horizon of the model, it was evident that results are sensitive to the time frame of the analysis (Figure 1). In both the US and the UK, PAV™ is associated with a lower cost of care in years 1 and 5. From year 10 onwards, PAV™ costs are higher than those with PSV. Given that all patients are in the home setting after 1 year, this indicates that it is the additional patient survival with PAV™ that is translating in to additional healthcare expenditure over the patient's life time. In terms of cost-effectiveness, PAV™ is dominant compared with PSV at a time horizon of 1 and 5 years, and would likely be considered cost-effective between time horizons of 10–40 years.

### Probabilistic sensitivity analyses

**Figure 2:**
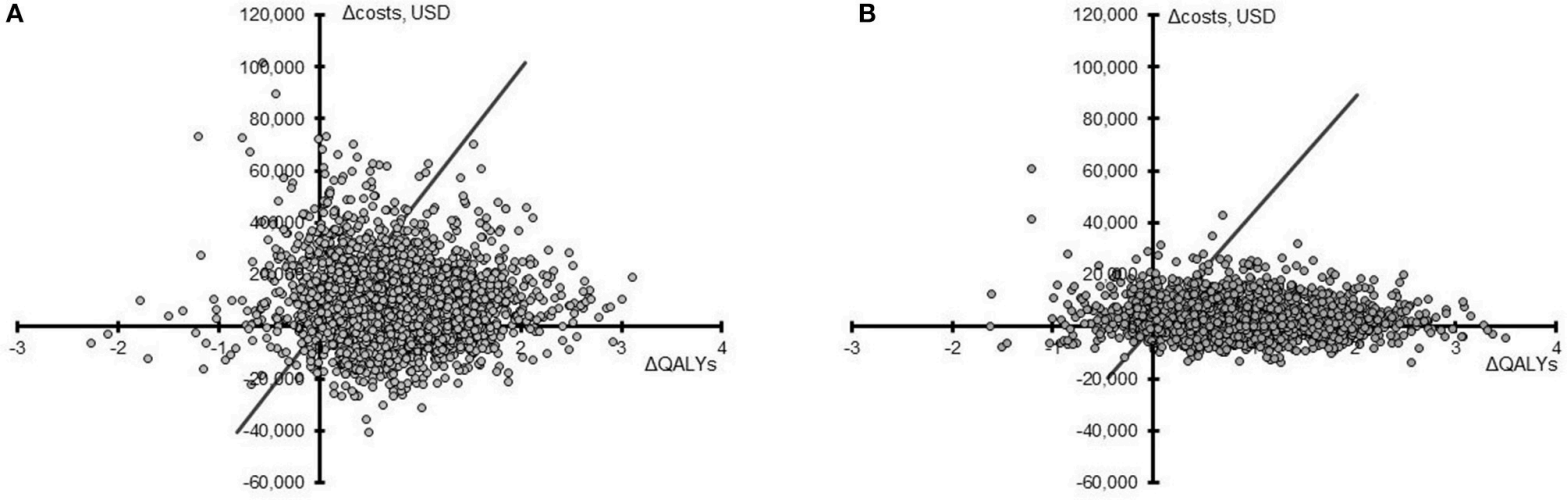
Cost-effectiveness plane for PAV™ relative to PSV. Results from 2,000 simulations in the US **(A)** and the UK **(B)** are presented as the difference in QALYs (X-axis) and the difference in total costs (Y-axis) for PAV™ vs. PSV (PAV™ minus PSV) over 40 years. The willingness-to-pay threshold of 50,000 USD or 30,000 GBP (converted here to USD to allow comparison) per QALY gained is shown by the diagonal line, with points under this line being considered cost-effective.

In assessing the impact of parameter uncertainty on model findings, 2,000 simulations were performed with random input values sampled from underlying distributions. In the majority of cases, results of these simulations were aligned with the base case. In the US setting, PAV™ increased life expectancy and QALYs in 89.40 and 89.25% of simulations, respectively, compared to PSV. The median QALY increase with PAV™ was 0.71 (95% CrI −0.47; 2.13). Costs were increased in 73.20% of cases, the median being USD 8,457 (95% CrI −17,813; 47,194). Overall, PAV™ would be considered cost-effective in 82.95% of simulations at a willingness-to-pay threshold of USD 50,000 per QALY gained (Figure 2). For the UK setting, similar results were obtained. PAV™ would be considered cost effective in 88.65% of simulations (Figure 2), with an increase in QALYs found in 90.75% of simulations (median 0.90, 95% CrI −0.51; 2.44). Costs with PAV™ were increased in 71.20% of simulations, median GBP 2,930 [$4,302] (95% CrI −7,709 [–$11,320]; 18,985 [$27,878]). As all CrIs at the 95%-level crossed zero, the differences are not considered significant. The trend in both countries is for improved patient outcomes with PAV™, and this is reflected in the substantial percentage of simulations in both settings in which PAV™ would be considered cost effective.

The results of these analyses provide a consistent picture of PAV™ being considered a cost-effective option for mechanical ventilation in the ICU over a 40-year time horizon in both the US and UK. Input data and their associated uncertainties were taken from individual publications, and these could vary considerably between individual hospitals and ICUs. For example, VAP incidence varied from 7.3 to 20.7 events per 1,000 ventilator-days in the UK (23). To test the impact of such variation, a series of scenario analyses were performed. The results, presented in Table 4, demonstrate that in most scenarios the findings from the analysis remain unchanged. The items having the most impact were the age of the patient population and the relative risks (RRs) associated with use of PAV™. If patients were aged 40 years on average, PAV™ remained cost-effective but the ICER increased. For patients aged 80 years on average, PAV™ dominated PSV in the UK setting and was very likely to be considered cost-effective in the US setting; potentially because annual healthcare costs were accumulated for fewer years in this older population. Similarly, if the RR of events with PAV™ (Table 2) was equal to that with PSV (RR 1.0), then PAV™ dominated PSV. This outcome could also be effected by switching only the RR of mortality to 1.0, again indicating the impact of life expectancy and future annual healthcare costs on outcomes. This item is further highlighted by the results in which the future annual care costs themselves were halved or eliminated. In contrast, the costs of the interventions and the incidence of adverse events did not substantially alter the findings of this analysis.

**Table 4:** The cost per QALY gained with PAV™ at 40 years is presented for a number of potential scenarios.

**Scenario**	**US**	**UK**
Patient population, age 40 years	$11,105	£1,443 [$2,119]
Patient population, age 80 years	$1,610	Dominant
VAP incidence, 20.7 per 1,000 ventilator days (35)	$9,014	£2,997 [$4,401]
VAP incidence, 7.3 per 1,000 ventilator days (35)	$9,028	£3,000 [$4,405]
Tracheostomy rate, 39.0% at day 20 (36)	$8,591	£2,978 [$4,373]
Annual care costs at 50%	$48	£137 [$201]
No annual care costs	Dominant	Dominant
PAV™ at an additional 4,000 currency units	$8,651	£3,017 [$4,430]
PAV™ at an additional 10,000 currency units	$8,686	£3,045 [$4,471]
PAV™ relative risks all set to 1, no difference from PSV	Dominant	Dominant
PAV™ time in state asynchrony >10%, 15.8% (doubled)	$8,811	£3,021 [$4,436]

*PAV™, proportional-assist ventilation with load-adjustable gain factors; PSV, Pressure support ventilation; VAP, Ventilator-associated pneumonia. Dominant, PAV™ is less costly than PSV and results in improved patient quality of life. USD values in square brackets are for comparative purposes and calculated using the mean annual exchange rate for USD:GBP in 2015*.

## Discussion

Mechanical ventilation via endotracheal tube is recognized as an important but costly intervention that helps maintain life in the critical care setting. There are several modes of mechanical ventilation, but one of the most common is PSV (19). Recent randomized, controlled trials have demonstrated that PAV™ has advantages over PSV (16, 17), but the question of cost-effectiveness has never been addressed. Our analysis determined that in both the US and UK settings, PAV™ is likely to be a cost-effective mode of mechanical ventilation compared with PSV. In both countries, PAV™ was associated with increased patient life expectancy and QALYs, as well as increased total costs of care over a 40-year time horizon. If a shorter time perspective was taken, then PAV™ may also be considered a cost saving intervention. Our analyses identified that differences in future costs of healthcare between the two interventions, driven by increased life expectancy with PAV™, was a key determinant of model outcomes.

As future annual care costs may be offset by insurance premium payments in the US and general taxation and national insurance payments in the UK, there is a reasonable question as to how these should be applied from a payer perspective. Guidelines from the National Institute for Health and Care Excellence (NICE) and Academy of Managed Care Pharmacy (AMCP) do not specify a time horizon to use (36, 37), but note that the time horizon implemented should be sufficient to capture “the period over which the main differences in health effects and use of healthcare resources between interventions are expected to be experienced” (36). As all patients are off mechanical ventilation and out of the hospital setting at 1 year, a short time horizon may be appropriate for decision makers. In fact, guidelines for economic analyses in the Netherlands do note that a lifelong time horizon is often inappropriate for evaluation of medical devices (38). In this setting, the minimum time horizon for evaluation of cost impact is specified at 3 years (38). Given uncertainty in the most appropriate time horizon to use for evaluation of medical devices, results are presented here after differing periods of time (Figure 1). If shorter time horizons are most appropriate to medical device decision making then it is important to note that in both countries at 1 and 5 years, PAV™ dominates PSV. Furthermore, in scenario analyses running for 40 years, halving or eliminating these future annual care costs resulted in PAV™ being considered highly cost-effective or dominant, respectively.

The results of the analyses were robust to changes in input parameters. The model is, however, only a representation of real life and based on average patients and mean outcome data. Clinical practice and even guidelines vary by country and hospital, the model cannot account for all these variations but aims to account for varying rates of events and intervention and levels of efficacy through sensitivity and scenario analyses. Results in individual hospitals would likely vary from those presented here because care practices, patient populations, and costs vary by institution. Furthermore, there is likely a learning curve to become familiar with the operation of PAV+ that has not and cannot (due to a lack of data) be explored in the model. The randomized, controlled trials informing this model were run by groups familiar with PAV+ and this may bias results relative to users new to the technology. Still, over a set of hospitals we would expect that results would be in line with those presented here. Probabilistic sensitivity analyses have shown that a payer would have a 95% chance of seeing a cost difference of between USD −17,813 and USD 47,194 in the US and GBP −7,709 [–$11,320] and GBP 18,985 [$27,878] in the UK, when a 40-year time horizon is used. The actual healthcare cost burden for a particular hospital using PAV™ will be dependent on local event rates and costs for devices, interventions, and personnel.

To fully validate the results of a model would require an extended, prospective cost collection study. This is beyond the realms of feasibility, and so model outputs are compared with previously reported values. In 2015, Hjelmgren et al. considered the cost consequence of neutrally-adjusted ventilatory assist (NAVA) mechanical ventilation relative to PSV in the Swedish setting (39). Their model varied substantially from that presented here, not considering future healthcare costs or any items outside of the ICU. Where comparable model outcomes were reported these were, however, in line with our analysis. Hjelmgren et al. reported a mean time on mechanical ventilation of 6.23–7.93 days (39), which is consistent with 6.77–6.97 days estimated here with use of PSV.

In 2012, Kollef et al. indicated that the cost of uncomplicated mechanical ventilation was USD 59,770 per patient (2). Over a single year in our model, PSV was at a cost of USD 60,008. This is closely aligned to the published value, and no cost data from Kollef et al. were used in our analysis. For the UK setting, Marti et al. reported total costs for survivors at GBP 19,195 (95% CI £15,936; £22,455) (5). Modeled outcomes at 1 year were within this range, being GBP 21,960 and GBP 19,749 for PSV and PAV™, respectively. As data presented by Marti et al., were used in our analysis this could have effected this association. Assessing the ICU stay with PSV, on average patients spent 9.11 days in the ICU, of which 6.77 were on a ventilator. The total costs for the ICU stay came to GBP 16,324, giving a mean cost of GBP 1,792 per day. As reported by Tan et al., the mean cost of an ICU day in the UK was EUR 2,025 in 2008 (3). Converting this to GBP using the mean annual exchange rate for 2008 (1.259 EUR = 1 GBP^2^), and then inflating it to 2015 pricing [using CPIH INDEX 06 (L528)] gave a value of GBP 1,940. The consistency between costing studies and our model results may help to validate the model structure and costing items included and provide more confidence in longer-term outcomes estimated.

Optimizing provision of mechanical ventilation is of high importance to healthcare providers. For payers, the costs of ICU care and mechanical ventilation in particular are a substantial burden on budgets. In general, an ICU accounts for fewer than 10% of hospital beds but over 20% of hospital costs (3). In a prospective study, ICU costs accounted for 64.7% of a patient's total hospital costs (40). The authors found, though, that per patient ICU costs reduced by 40% between 2008 and 2011, although the daily ICU cost decreased by only 3.3% in this time (40). Reducing the cost burden of ICU care is therefore possible without reducing direct costs, but rather through optimization of care. Multivariate analysis determined that fewer nosocomial infection and reduced ICU length of stay were two of six significant factors reducing ICU costs (40). Our analysis has demonstrated that use of PAV™, relative to PSV, can reduce patient-ventilator asynchrony and decrease time on mechanical ventilation and in the ICU. Aligned with previous studies, this resulted in a lower cost burden for ICU care. Over the long term, general healthcare (non-ICU) costs may increase due to improved life expectancy.

From a provider perspective, use of PAV™ could result in lower resource use and improved patient comfort and quality of life. A key aspect of the PAV™ technology is the reduction in patient-ventilator asynchrony. Scenario analyses changing the relative level of asynchrony did not, however, have a large impact on cost-effectiveness outcomes. More pertinent were fewer days on mechanical ventilation and in the ICU, as reported by Bosma et al. (16) This was linked to other model results, such as lower incidence of nosocomial infections and reduced use of tracheostomy with PAV™. As in the randomized, controlled trials reporting on these outcomes, these differences in the model did not reach significance. To confirm the potential cost and health benefits of PAV™ identified in this analysis would require large scale clinical and cost collection studies. To date, the two randomized, controlled trials of PAV™ have enrolled 262 patients and no costs have been reported (16, 17). The implication for improved care they provide is important, particularly as larger studies may impinge on clinician duties and may not be feasible on the scale required. To this end, computational models are one option available to combine data from multiple sources and inform the debate on best practice in the ICU. The presented clinical model finds that PAV™ is likely to be considered cost-effective in the US and UK settings. The validity of the data is supported by the fact that the results are in line with published cost and outcome data not used to inform the model development.

## Conclusion

The published reductions in asynchrony and length of stay in the ICU with PAV™ (16, 17). led to shorter time on ventilation, and reduced incidence of VAP and tracheostomy in this decision analytic model. Increased patient survival with PAV™ resulted in annual healthcare costs being accumulated over a longer period. This made it most likely that PAV™ is cost-saving in the short term and cost-effective over the long term. For payers, PAV+ is likely a cost-effective option for mechanical ventilation when compared with PSV. For hospitals, PAV™ is expected to reduce costs and resource use.

## Author contributions

DG provided information on the patient care pathway and clinical and economics outcomes to include. RS performed the literature search and review and created the initial model design. The model was finalized after review and updates from DG. RS ran analyses and sensitivity analyses and drafted the manuscript. Manuscript review and updates were performed by DG. Both authors met to approve the final manuscript for submission.

### Conflict of interest statement

RS is the owner of Coreva Scientific GmbH & Co KG, which received consultancy fees for performing, analysing, and communicating the work presented here. DG received an honorarium from Medtronic Inc. for identifying clinical data and helping to conceptualize the clinical model developed.

## Footnotes

^1^Annual exchange rate for 2015 from the United States of America Inland Revenue Service (https://www.irs.gov/individuals/international-taxpayers/yearly-average-currency-exchange-rates) accessed 17 May 2018.

^2^*Mean Exchange rate from www.x-rates.com*
