# Supplementary material for: Evaluating the Cost-Effectiveness of Proportional-Assist Ventilation Plus vs. Pressure Support Ventilation in the Intensive Care Unit in Two Countries
Source: Front Public Health. 2018 Jun 6;6:168. doi: 10.3389/fpubh.2018.00168 (PMC5998768; doi:10.3389/fpubh.2018.00168)
Supplement: Supplementary file 1 [file Data_Sheet_1.docx]

# Supplement

## Cost data

The aim of this literature search was to identify recent (published since 2012) literature reporting on the costs associated with mechanical ventilation, tracheotomy, and ventilator-associated pneumonia. The literature search in PubMed was performed on January 05, 2017.

Table S1 Structured searches in PubMed to identify relevant cost data

| Index | Aim | Search string | Hits |
| --- | --- | --- | --- |
| 1 | All cost studies | "Costs and Cost Analysis"[Mesh] OR "Cost-Benefit Analysis"[Mesh] OR "Cost of Illness"[Mesh] OR "Health Care Costs"[Mesh] OR "Cost Sharing"[Mesh] OR "Cost Savings"[Mesh] OR "Technology, High-Cost"[Mesh] OR "Cost Control"[Mesh] OR "Cost Allocation"[Mesh] OR "Direct Service Costs"[Mesh] OR "Hospital Costs"[Mesh] OR "Employer Health Costs"[Mesh] OR "Drug Costs"[Mesh] OR "Health Expenditures"[Mesh] OR "Health Resources/economics"[Mesh] OR "Economics, Hospital"[Mesh] OR "Economics, Medical"[Mesh] OR "Economics, Pharmaceutical"[Mesh] OR "Economics, Nursing"[Mesh] OR "Managed Care Programs"[Mesh] OR "Insurance, Physician Services"[Mesh] OR "Budgets"[Mesh] OR "Economics"[Mesh] OR "Commerce"[Mesh] OR Cost[tiab] OR economic[tiab] OR ((EURO[tiab] OR EUROS[tiab] OR GBP[tiab] OR USD[tiab] OR dollar*[tiab] OR pounds[tiab]) AND (Cost[tiab] OR price[tiab] or expense[tiab] OR burden[tiab])) | 884,505 |
| 2 | All studies since 2012 | "2012/01/01"[PDAT]: "2017/01/01"[PDAT] | 5,322,536 |
| 3 | Recent cost studies | #1 AND #2 | 218,488 |
| 4 | Adverse events (AE) of interest | "Tracheotomy"[Mesh] OR "Tracheotomy"[tiab] OR "Tracheostomy"[tiab] OR "Pneumonia, Ventilator-Associated"[Mesh] OR VAP[tiab] OR "ventilator-associated pneumonia"[tiab] OR "Respiration, Artificial/adverse effects"[Mesh] OR "Respiration, Artificial/complications"[Mesh] OR "Respiration,  Artificial/economics"[Mesh] OR "Respiration, Artificial/mortality"[Mesh] OR "Respiration, Artificial/statistics and numerical data"[Mesh] OR "Length of Stay/economics"[Mesh] OR synchrony[tiab] OR synchronous[tiab] OR asynchrony[tiab] OR asynchronous[tiab] OR ((LOS[tiab] OR stay[tiab]) AND (ICU[tiab] OR "intensive care"[tiab])) OR ((mortality[tiab] OR death[tiab] OR surviving[tiab] OR survival[tiab]) AND (ICU[tiab] OR "intensive care"[tiab] or "critically ill"[tiab])) | 138,041 |
| 5 | Those reporting on assisted ventilation | "Respiration, Artificial"[Mesh] OR "High-Frequency Ventilation"[Mesh] OR "Interactive Ventilatory Support"[Mesh] OR "mechanical ventilation"[tiab] OR "assisted ventilation"[tiab] OR "proportional assist"[tiab] OR PAV[tiab] OR “PAV+™”[tiab] OR NAVA[tiab] OR PSV[tiab] OR "neurally adjusted"[tiab] OR "artificial respiration"[tiab] | 86,497 |
| 6 | AEs and assisted ventilation | #4 AND #5 | 22,818 |
| 7 | Cost of assisted ventilation | #3 AND #6 | 407 |
| 8 | Avoid non-primary research | "Case Reports" [Publication Type] OR "Clinical Conference" [Publication Type] OR "Comment" [Publication Type] OR "Editorial" [Publication Type] OR "Guideline" [Publication Type] OR "Review" [Publication Type] | 4,841,567 |
| 9 | Relevant studies | #7 NOT #8 | 318 |
| 10 | Containing abstracts | hasabstract[text] | 16,930,518 |
| 11 | Language | english[lang] | 22,253,658 |
| 12 | Animal studies | (see Section 6) | 6,261,153 |
| 13 | Final reference list | #9 AND # 10 AND # 11 NOT # 12 | 286 |

## Efficacy and safety

The aim of this literature search was to identify recent (published since 2007) literature reporting on RCTs associated with PAV+™ technology. The literature search in PubMed was performed on January 05, 2017.

Table S2 Structured searches in PubMed to identify efficacy data from randomized, controlled, clinical trials and meta-analyses

| Index | Aim | Search string | Hits |
| --- | --- | --- | --- |
| 1 | Those reporting on assisted ventilation | "Respiration, Artificial"[Mesh] OR "High-Frequency Ventilation"[Mesh] OR "Interactive Ventilatory Support"[Mesh] OR "mechanical ventilation"[tiab] OR "assisted ventilation"[tiab] OR "proportional assist"[tiab] OR PAV[tiab] OR “PAV+™”[tiab] OR NAVA[tiab] OR PSV[tiab] OR "neurally adjusted"[tiab] OR "artificial respiration"[tiab] | 86,601 |
| 2 | Those specific to asynchrony? | weaning[tiab] or synchrony[tiab] OR synchronies[tiab] or synchronous[tiab] OR asynchrony[tiab] or asynchronies[tiab] OR asynchronous[tiab] or "ineffective efforts"[tiab] | 72,548 |
| 3 | Only in intensive care unit | ICU[tiab] OR "intensive care"[tiab] or "critically ill"[tiab] | 144,178 |
| 4 | All studies since 2012 | "2007/01/01"[PDAT]: "2017/01/01"[PDAT] | 9,281,870 |
| 5 | Recent studies | #1 AND #2 AND #3 AND #4 | 975 |
| 6 | Those reporting on Randomized controlled trials (RCT) | "prospective observational"[tiab] or "Controlled Clinical Trial" [Publication Type] or (("Clinical Trial"[tiab] or "Clinical study"[tiab] or "Clinical studies"[tiab] or "Clinical Trials"[tiab]) AND (randomized[tiab] AND (blinded[tiab] OR controlled[tiab])) or "Randomized Controlled Trials as Topic"[Mesh] OR "Clinical Trial"[Publication Type] OR Randomized Controlled Trial[tiab] or RCT[tiab] OR "Comparative Study"[Publication Type] OR "Meta-Analysis"[Publication Type] OR meta-analysis[tiab] OR (indirect[tiab] AND “treatment comparison”[tiab]) | 2,515,820 |
| 7 | Studies of interest | #5 AND #6 | 284 |
| 8 | Avoid non-primary research | "Case Reports" [Publication Type] OR "Clinical Conference" [Publication Type] OR "Comment" [Publication Type] OR "Editorial" [Publication Type] OR "Guideline" [Publication Type] OR "Review" [Publication Type] | 4,846,604 |
| 9 | Relevant studies | #7 NOT #8 | 244 |
| 8 | Containing abstracts | hasabstract[text] | 16,954,684 |
| 9 | Language | english[lang] | 22,281,256 |
| 10 | Animal studies | (see Section 6) | 6,265,837 |
| 11 | Final reference list | #7 AND # 8 AND # 9 NOT # 10 | 224 |

## Health utility data

The aim of this literature search was to identify recent (published since 2012) literature reporting on the quality of life associated with PAV+™ technology. The literature search in PubMed was performed on January 05, 2017.

Table S3 Structured searches in PubMed to identify health state utility and disutility relevant to modelling

| Index | Aim | Search string | Hits |
| --- | --- | --- | --- |
| 1 | All quality of life studies | (("Quality of Life"[Mesh] OR EuroQol[tiab] OR EQ5D[tiab] OR SF36[tiab] OR ((“short-form”[tiab] OR “short form”[tiab]) AND (survey[tiab] OR questionnaire[tiab])) OR ((“health-related”[tiab] OR “health related”[tiab] OR “health state”[tiab] OR “health-state”[tiab]) AND (utility[tiab] OR utilities[tiab] OR disutility[tiab] OR disutilities[tiab] OR "quality of life"[tiab] OR QOL[tiab])) OR “patient preference”[tiab] OR “patient satisfaction”[tiab])) | 188,494 |
| 2 | All studies since 2012 | "2012/01/01"[PDAT]: "2017/01/01"[PDAT] | 5,322,536 |
| 3 | Recent quality of life studies | #1 AND #2 | 66,939 |
| 4 | Those reporting on assisted ventilation | "Respiration, Artificial"[Mesh] OR "High-Frequency Ventilation"[Mesh] OR "Interactive Ventilatory Support"[Mesh] OR "mechanical ventilation"[tiab] OR "assisted ventilation"[tiab] OR "proportional assist"[tiab] OR PAV[tiab] OR “PAV+™”[tiab] OR NAVA[tiab] OR PSV[tiab] OR "neurally adjusted"[tiab] OR "artificial respiration"[tiab] OR VAP[tiab] OR "ventilator-associated pneumonia"[tiab] or "ventilator associated pneumonia"[tiab] OR "Tracheotomy"[Mesh] OR "Tracheotomy"[tiab] OR "Tracheostomy"[tiab] OR "Pneumonia, Ventilator-Associated"[Mesh] OR asynchrony[tiab] OR asynchronous[tiab] OR nocosomial[tiab] | 117,003 |
| 5 | Pediatrics and home mechanical ventilation excluded | pediatric[ti] or paediatric[ti] OR neonatal[ti] OR neonates[ti] OR children[ti] OR child[ti] OR home[tiab] | 879,352 |
| 6 | Relevant studies | #3 AND #4 NOT #5 | 382 |
| 7 | Avoid non-primary research | "Case Reports" [Publication Type] OR "Clinical Conference" [Publication Type] OR "Comment" [Publication Type] OR "Editorial" [Publication Type] OR "Guideline" [Publication Type] OR "Review" [Publication Type] | 4,841,567 |
| 8 |  | #6 NOT #7 | 271 |
| 9 | Containing abstracts | hasabstract[text] | 16,930,518 |
| 10 | Language | english[lang] | 22,253,658 |
| 11 | Animal studies | (see Section 6) | 6,261,153 |
| 12 | Final reference list | #8 AND # 9 AND # 10 NOT # 11 | 244 |

## Model diagram

Figure S1 Representation of patient progression through the model


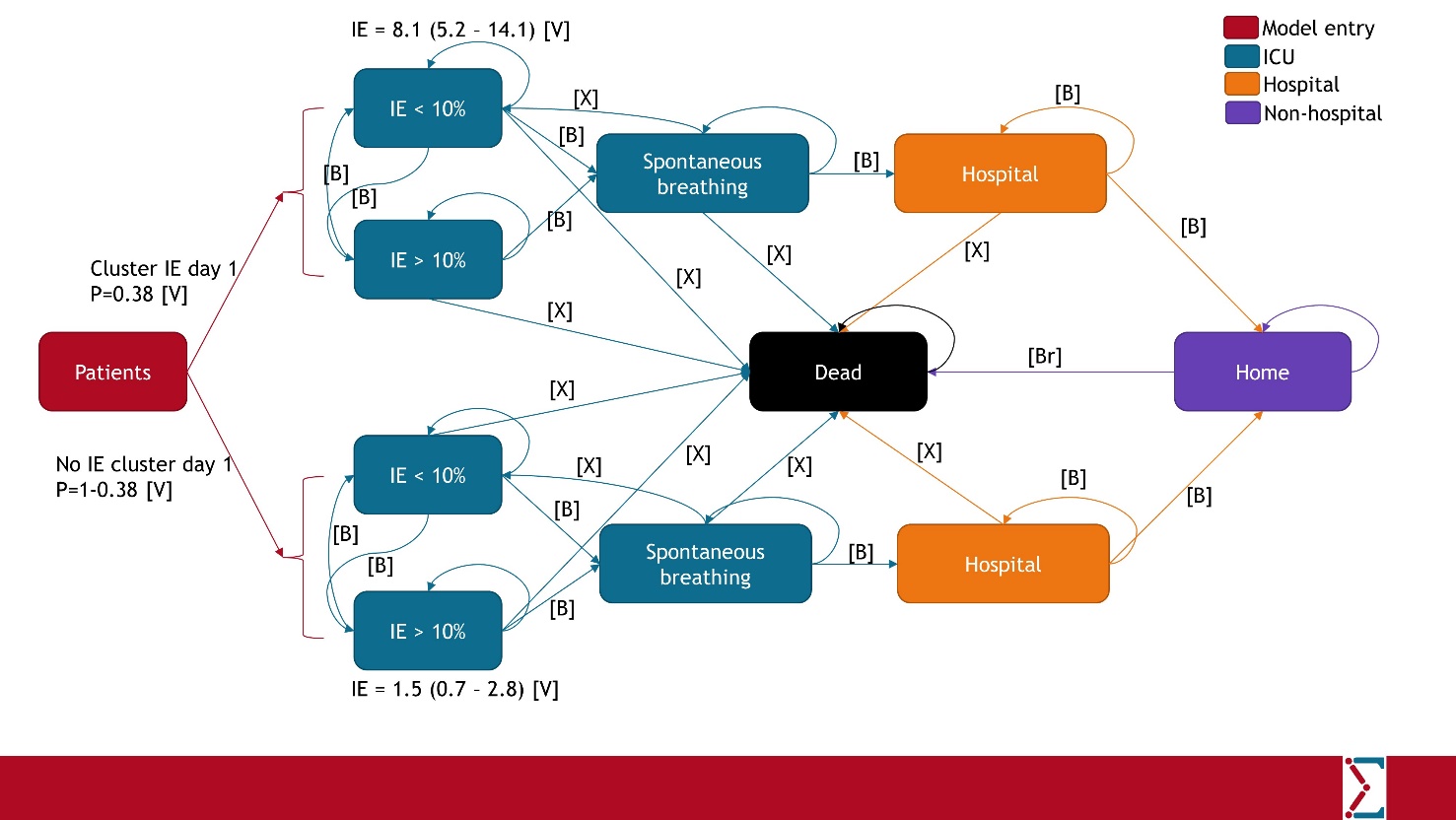


The model takes a cohort of patients that commence mechanical ventilation (MV). In this description we will discuss “patient(s)”, but in the model it is the probability of the patient being in that situation at the given time. Presented in this figure is the representation of the patient pathway, with the ICU containing health states of mechanical ventilation with asynchrony (IE > 10%), of mechanical ventilation without asynchrony (IE < 10%) and spontaneous breathing trials (spontaneous breathing). Once a patient passes the spontaneous breathing trial they can be released from the ICU to the general ward (hospital) where they remain until discharge. After discharge the patient is in the home setting. At all stages the patient may die, with the probability of death related to the patient’s position in the pathway, i.e. rates of death specific to MV, the ICU, hospital post-ICU, and home post-MV. The model is run twice, once for PAV+™ and once for the competitor product, the outcomes of the two runs are then compared. For each product, PAV+™ or competitor, patients enter either the upper or lower model, with those in the upper model having had clusters of ineffectual efforts on initiation of MV. These patients are more likely to have a higher IE (asynchrony index [AI) index and have increased risk of mortality. Transition probabilities are informed by randomized, clinical trials; prospective studies, and other peer-reviewed published literature.[B]=Bosma[1]; [Br]=Brinkman[2]; [V]=Vaporidi[3]; [X]=Xirouchaki[4].

**References**

[1] Bosma KJ, Read BA, Bahrgard Nikoo MJ, Jones PM, Priestap FA, Lewis JF. A Pilot Randomized Trial Comparing Weaning From Mechanical Ventilation on Pressure Support Versus Proportional Assist Ventilation. Crit Care Med 2016;44:1–11. doi:10.1097/CCM.0000000000001600.

[2] Brinkman S, de Jonge E, Abu-Hanna A, Arbous MS, de Lange DW, de Keizer NF. Mortality after hospital discharge in ICU patients. Crit Care Med 2013;41:1229–36. doi:10.1097/CCM.0b013e31827ca4e1.

[3] Vaporidi K, Babalis D, Chytas A, Lilitsis E, Kondili E, Amargianitakis V, et al. Clusters of ineffective efforts during mechanical ventilation: impact on outcome. Intensive Care Med 2017;43:184–91. doi:10.1007/s00134-016-4593-z.

[4] Xirouchaki N, Kondili E, Vaporidi K, Xirouchakis G, Klimathianaki M, Gavriilidis G, et al. Proportional assist ventilation with load-adjustable gain factors in critically ill patients: Comparison with pressure support. Intensive Care Med 2008;34:2026–34. doi:10.1007/s00134-008-1209-2.
